# Supplementary figures and images for: Evaluation and Transcriptome Analysis of the Novel Oleaginous Microalga Lobosphaera bisecta (Trebouxiophyceae, Chlorophyta) for Arachidonic Acid Production
Source: Mar Drugs. 2020 Apr 26;18(5):229. doi: 10.3390/md18050229 (PMC7281613; doi:10.3390/md18050229)

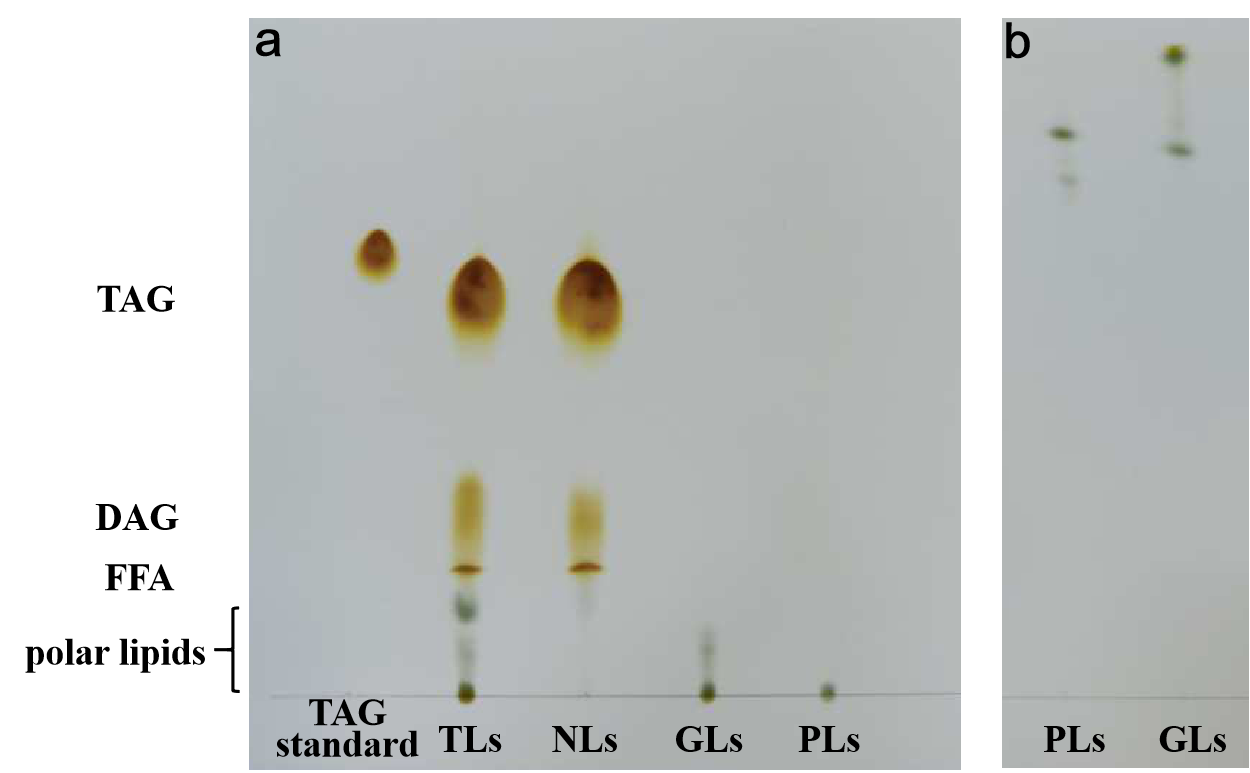

Supplement: Supplementary file 1 [file marinedrugs-18-00229-s001.zip › Supplementary File/Figure S1.tif]
