# Supplementary material for: Evaluation and Transcriptome Analysis of the Novel Oleaginous Microalga Lobosphaera bisecta (Trebouxiophyceae, Chlorophyta) for Arachidonic Acid Production
Source: Mar Drugs. 2020 Apr 26;18(5):229. doi: 10.3390/md18050229 (PMC7281613; doi:10.3390/md18050229)
Supplement: Supplementary file 1 [file marinedrugs-18-00229-s001.zip › Supplementary File/Table S3.doc]

Table S1. The raw data generated and mapping on the reference transcriptome.

| Sample | Read number (bp) | | Base number (bp) | | GC content (%) | | Q30  (%) | | Mapped Reads  (bp) | | Mapped Ratio (%) | |
| --- | --- | --- | --- | --- | --- | --- | --- | --- | --- | --- | --- | --- |
| SAG2043-NR | | 26,988,800 | | 8,080,864,474 | | 59.08 | | 92.26 | | 21,048,364 | | 77.99 |
| SAG2043-NF | | 29,166,965 | | 8,730,259,012 | | 59.79 | | 92.50 | | 24,061,072 | | 82.49 |
