# Supplementary material for: Evaluation and Transcriptome Analysis of the Novel Oleaginous Microalga Lobosphaera bisecta (Trebouxiophyceae, Chlorophyta) for Arachidonic Acid Production
Source: Mar Drugs. 2020 Apr 26;18(5):229. doi: 10.3390/md18050229 (PMC7281613; doi:10.3390/md18050229)
Supplement: Supplementary file 1 [file marinedrugs-18-00229-s001.zip › Supplementary File/Table S5.doc]

Table S5. Primers for genes by Quantitative real-time polymerase chain reaction (RT-qPCR)

| Genes | Unigene ID | Primer | Sequence (5'–3′) | Product size (bp) |
| --- | --- | --- | --- | --- |
| stearoyl-acp 9-desaturase (**9 des**) | Unigene_BMK.6557 | Desa9-F | GCCTGCCCGACGACTACCTG | 168 |
|  |  | Desa9-R | GGTTCTCCTCGGCCGTCCAC |  |
| Oleoyl-acp thioesterase (**thioe**) | Unigene_BMK.38890 | Thioes-F | TCCCAGCAATCCGCAGCGTT | 133 |
|  |  | Thioes-R | ATGGCGTCCTGACCCGTCCT |  |
| 3-ketoacyl-ACP-synthase II (**KASII**) | Unigene_BMK.10266 | KAS-F | TGCTCACACAACGAGGAGAG | 121 |
|  |  | KAS-R | TTGCCATCTGGACCGCTATG |  |
| delta 5 desaturase (**5 des**) | Unigene_BMK.3691 | Desa5-F | CGCGTGAAGGACCCCGATGT | 196 |
|  |  | Desa5-R | CCTTCTCCCCAGGCGTCAGC |  |
| delta 6 desaturase (**6 des**) | Unigene_BMK.39610 | Desa6-F | GTGATCTACACCTACGCGGG | 105 |
|  |  | Desa6-R | CACCACATCCCCAACATGGA |  |
| 18S rRNA |  | 18S-F | ACGGTGAAACTGCGAATGGCT | 195 |
|  |  | 18S-R | GGCCGTGCGATTCGTGAAGT |  |
