# Supplementary material for: Evaluation and Transcriptome Analysis of the Novel Oleaginous Microalga Lobosphaera bisecta (Trebouxiophyceae, Chlorophyta) for Arachidonic Acid Production
Source: Mar Drugs. 2020 Apr 26;18(5):229. doi: 10.3390/md18050229 (PMC7281613; doi:10.3390/md18050229)
Supplement: Supplementary file 1 [file marinedrugs-18-00229-s001.zip › Supplementary File/table S1, S2.doc]

**Table S1.** Change in fatty acid content (% total fatty acids) of *L. bisecta* SAG2043 with a high concentration of nitrogen (18 mM) under medium replacement and bilateral high light intensity (bl-300) cultivation over a time course. 18 mM-18 mM, medium replacement from initial nitrogen concentration of 18 mM to fresh medium of 18 mM on day 9; 18 mM-0 mM, medium replacement from initial nitrogen concentration of 18 mM to fresh medium of 0 mM on day 9.

|  | **18 mM-18 mM** | | | | | | | | **18 mM-0 mM** | | | | |
| --- | --- | --- | --- | --- | --- | --- | --- | --- | --- | --- | --- | --- | --- |
| **day** | **3** | **6** | **9** | **12** | **15** | **18** | **21** | **24** | **12** | **15** | **18** | **21** | **24** |
| **C16:0** | **14.68±**  **1.45** | **14.37±**  **1.33** | **12.69±**  **1.49** | **12.96±**  **0.82** | **12.07±**  **0.55** | **11.11±**  **1.20** | **10.60±**  **0.47** | **10.28±**  **0.82** | **11.41±**  **0.66** | **10.06±**  **0.38** | **9.68±**  **1.17** | **9.40±**  **1.37** | **9.14±**  **0.92** |
| **C18:0** | **0.27±**  **0.10** | **0.74±**  **0.48** | **1.22±**  **0.38** | **0.89±**  **0.25** | **1.15±**  **0.11** | **1.30±**  **0.41** | **1.44±**  **0.42** | **1.46±**  **0.55** | **1.38±**  **0.69** | **1.80±**  **0.81** | **2.02±**  **0.72** | **2.14±**  **0.84** | **2.11±**  **0.66** |
| **C18:1** | **15.23±**  **1.17** | **19.35±**  **1.24** | **25.02±**  **2.21** | **22.85±**  **1.48** | **28.80±**  **1.34** | **33.65±**  **2.50** | **36.11±**  **2.03** | **37.32±**  **2.21** | **32.59±**  **1.67** | **36.26±**  **1.77** | **38.74±**  **2.24** | **39.38±**  **2.35** | **39.65±**  **2.49** |
| **C18:2** | **13.25±**  **1.24** | **18.14±**  **0.79** | **16.82±**  **1.90** | **18.92±**  **1.13** | **17.33±**  **1.48** | **16.23±**  **1.67** | **15.27±**  **1.24** | **14.61±**  **0.83** | **14.81±**  **1.06** | **14.54±**  **0.75** | **14.79±**  **1.16** | **14.80±**  **1.62** | **14.84±**  **0.83** |
| **C18:3** | **16.06±**  **1.49** | **11.79±**  **0.55** | **6.94±**  **0.69** | **6.92±**  **0.73** | **5.39±**  **0.49** | **4.40±**  **0.80** | **3.89±**  **0.28** | **3.40±**  **0.64** | **5.57±**  **0.31** | **4.42±**  **1.05** | **3.98±**  **0.93** | **3.69±**  **0.49** | **3.40±**  **0.57** |
| **C20:4** | **12.94±**  **1.13** | **12.61±**  **0.86** | **16.10±**  **1.34** | **19.48±**  **1.57** | **20.83±**  **1.68** | **21.69±**  **1.82** | **22.72±**  **1.20** | **23.62±**  **1.63** | **21.45±**  **0.99** | **22.42±**  **1.60** | **22.52±**  **1.43** | **22.95±**  **1.74** | **22.92±**  **1.63** |
| **C20:5** | **2.80±**  **0.34** | **3.00±**  **0.61** | **2.49±**  **0.53** | **2.65±**  **0.48** | **2.28±**  **0.67** | **1.96±**  **0.45** | **1.84±**  **0.18** | **1.71±**  **0.34** | **2.22±**  **0.63** | **1.90±**  **0.44** | **1.74±**  **0.31** | **1.68±**  **0.52** | **1.67±**  **0.29** |
| **SFA** | **14.95±**  **1.33** | **15.11±**  **0.87** | **13.91±**  **0.63** | **13.85±**  **1.29** | **13.22±**  **1.50** | **12.41±**  **1.73** | **12.04±**  **1.54** | **11.74±**  **1.62** | **12.79±**  **0.73** | **11.86±**  **0.91** | **11.7±**  **0.77** | **11.54±**  **1.26** | **11.25±**  **1.49** |
| **MUFA** | **15.23±**  **1.17** | **19.35±**  **1.24** | **25.02±**  **2.21** | **22.85±**  **1.48** | **28.80±**  **1.34** | **33.65±**  **2.50** | **36.11±**  **2.03** | **37.32±**  **2.21** | **32.59±**  **1.67** | **36.26±**  **1.77** | **38.74±**  **2.24** | **39.38±**  **2.35** | **39.65±**  **2.49** |
| **PUFA** | **45.05±**  **2.11** | **45.54±**  **2.30** | **42.35±**  **2.57** | **47.97±**  **2.66** | **45.83±**  **2.34** | **44.28±**  **1.52** | **43.72±**  **1.44** | **43.34±**  **2.03** | **44.05±**  **1.88** | **43.28±**  **1.16** | **43.03±**  **1.28** | **43.12±**  **2.21** | **42.83±**  **3.07** |

**Table S2**. Change in fatty acid content (% total fatty acids) of *L. bisecta* SAG2043 with a low concentration of nitrogen (3.6 mM) under medium replacement and bilateral high light intensity (bl-300) cultivation over a time course. 3.6 mM-3.6 mM, medium replacement from initial nitrogen concentration of 3.6 mM to fresh medium of 3.6 mM on day 9; 3.6 mM-0 mM, medium replacement from initial nitrogen concentration of 3.6 mM to fresh medium of 0 mM on day 9.

|  | **3.6 mM-3.6 mM** | | | | | | | | **3.6 mM-0 mM** | | | | |
| --- | --- | --- | --- | --- | --- | --- | --- | --- | --- | --- | --- | --- | --- |
| **day** | **3** | **6** | **9** | **12** | **15** | **18** | **21** | **24** | **12** | **15** | **18** | **21** | **24** |
| **C16:0** | **15.94±**  **1.82** | **13.16±**  **1.54** | **10.77±**  **1.35** | **11.46±**  **0.69** | **10.52±**  **0.75** | **9.76±**  **1.20** | **9.55±**  **0.64** | **9.59±**  **0.77** | **9.69±**  **0.86** | **9.10±**  **0.94** | **8.94±**  **1.08** | **8.91±**  **1.30** | **8.96±**  **0.51** |
| **C18:0** | **0.56±**  **0.22** | **1.42±**  **0.37** | **1.18±**  **0.50** | **1.73±**  **0.59** | **2.02±**  **0.43** | **2.04±**  **0.75** | **1.79±**  **0.53** | **1.94±**  **0.40** | **1.19±**  **0.08** | **2.33±**  **0.63** | **2.03±**  **0.58** | **2.30±**  **0.63** | **2.25±**  **0.46** |
| **C18:1** | **17.62±**  **1.49** | **34.27±**  **2.08** | **42.69±**  **2.27** | **37.79±**  **2.41** | **39.69±**  **2.38** | **41.14±**  **1.42** | **41.28±**  **2.04** | **41.22±**  **1.64** | **44.95±**  **1.46** | **44.08±**  **2.31** | **43.14±**  **1.81** | **42.68±**  **1.66** | **42.12±**  **1.75** |
| **C18:2** | **14.59±**  **1.63** | **13.68±**  **1.40** | **13.45±**  **1.08** | **13.17±**  **0.94** | **13.02±**  **1.33** | **12.87±**  **1.21** | **12.92±**  **1.54** | **13.14±**  **0.96** | **12.13±**  **1.24** | **11.89±**  **1.53** | **11.70±**  **0.84** | **11.81±**  **0.67** | **11.95±**  **0.55** |
| **C18:3** | **15.81±**  **0.86** | **7.95±**  **1.32** | **5.41±**  **1.08** | **5.75±**  **0.63** | **4.54±**  **0.54** | **3.92±**  **0.37** | **3.64±**  **0.58** | **3.45±**  **0.39** | **4.51±**  **0.43** | **3.92±**  **0.91** | **3.61±**  **0.18** | **3.42±**  **0.50** | **3.27±**  **0.76** |
| **C20:4** | **14.14±**  **1.63** | **18.38±**  **1.28** | **18.58±**  **1.63** | **22.33±**  **1.55** | **22.83±**  **1.27** | **23.18±**  **2.30** | **23.63±**  **1.08** | **24.02±**  **2.11** | **21.04±**  **1.54** | **22.56±**  **1.27** | **23.34±**  **1.36** | **24.79±**  **1.09** | **25.32±**  **1.18** |
| **C20:5** | **3.57±**  **0.82** | **2.80±**  **0.75** | **2.05±**  **0.23** | **2.61±**  **0.34** | **2.27±**  **0.63** | **2.04±**  **0.74** | **1.96±**  **0.68** | **1.92±**  **0.54** | **1.83±**  **0.73** | **1.70±**  **0.49** | **1.67±**  **0.31** | **1.68±**  **0.27** | **1.71±**  **0.59** |
| **SFA** | **16.5±**  **1.08** | **14.58±**  **1.55** | **11.95±**  **1.60** | **13.19±**  **1.33** | **12.54±**  **0.59** | **11.8±**  **0.67** | **11.34±**  **1.24** | **11.53±**  **1.48** | **10.88±**  **1.61** | **11.43±**  **0.88** | **10.97±**  **0.67** | **11.21±**  **1.54** | **11.21±**  **0.62** |
| **MUFA** | **17.62±**  **1.49** | **34.27±**  **2.08** | **42.69±**  **2.27** | **37.79±**  **2.41** | **39.69±**  **2.38** | **41.14±**  **1.42** | **41.28±**  **2.04** | **41.22±**  **1.64** | **44.95±**  **1.46** | **44.08±**  **2.31** | **43.14±**  **1.81** | **42.68±**  **1.66** | **42.12±**  **1.75** |
| **PUFA** | **48.11±**  **2.13** | **42.81±**  **2.25** | **39.49±**  **2.77** | **43.86±**  **2.61** | **42.66±**  **2.84** | **42.01±**  **2.30** | **42.15±**  **2.66** | **42.53±**  **2.07** | **39.51±**  **1.59** | **40.07±**  **2.74** | **40.32±**  **1.11** | **41.70±**  **1.67** | **42.25±**  **3.01** |
